# Impact of the Reference Multiple-Time-Point Dosimetry Protocol on the Validity of Single-Time-Point Dosimetry for [^177^Lu]Lu-PSMA-I&T Therapy

**DOI:** 10.2967/jnumed.123.266871

**Published:** 2024-08

**Authors:** Sandra Resch, Sibylle I. Ziegler, Gabriel Sheikh, Lena M. Unterrainer, Mathias J. Zacherl, Peter Bartenstein, Guido Böning, Julia Brosch-Lenz, Astrid Delker

**Affiliations:** 1 Department of Nuclear Medicine, LMU University Hospital, LMU, Munich, Germany;; 2 Ahmanson Translational Theranostics Division, Department of Molecular and Medical Pharmacology, UCLA, Los Angeles, California; and; 3 Department of Nuclear Medicine, Technical University of Munich, Munich, Germany

**Keywords:** PSMA, ^177^Lu, dosimetry, single time point

## Abstract

Internal dosimetry supports safe and effective patient management during radionuclide therapy. Yet, it is associated with high clinical workload, costs, and patient burden, as patient scans at multiple time points (MTPs) must be acquired. Dosimetry based on imaging at a single time point (STP) has continuously gained popularity. However, MTP protocols, used as a reference to judge the validity of STP dosimetry, differ depending on local requirements and deviate from the unknown patient-specific ground truth pharmacokinetics. The aim of this study was to compare the error and optimum time point for different STP approaches using different reference MTP protocols. **Methods:** Whole-body SPECT/CT scans of 7 patients (7.4–8.9 GBq of [^177^Lu]Lu-PSMA-I&T) were scheduled at 24, 48, 72, and 168 h after injection. Sixty lesions, 14 kidneys, and 10 submandibular glands were delineated in the SPECT/CT data. Two curve models, that is, a mono- and a biexponential model, were fitted to the MTP data, in accordance with goodness-of-fit analysis (coefficients of variation, sum of squared errors). Three population-based STP approaches were compared: one method published by Hänscheid et al., one by Jackson et al., and one using population-based effective half-lives in the mono- or biexponential curve models. Percentage differences between STP and MTP dosimetry were evaluated. **Results:** Goodness-of-fit parameters show that a monoexponential function and a biexponential function with shared population-based parameters and physical tail are reasonable reference models. When comparing both reference models, we observed maximum differences of −44%, −19%, and −28% in the estimated absorbed doses for lesions, kidneys, and salivary glands, respectively. STP dosimetry with an average deviation of less than 10% from MTP dosimetry may be feasible; however, this deviation and the optimum imaging time point showed a dependence on the chosen reference protocol. **Conclusion:** STP dosimetry for [^177^Lu]Lu-PSMA therapy is promising to boost the integration of dosimetry into clinical routine. According to our patient cohort, 48 h after injection may be regarded as a compromise for STP dosimetry for lesions and at-risk organs. The results from this analysis show that a common gold standard for dosimetry is desirable to allow for reliable and comparable STP dosimetry.

## Visual Abstract

**Figure:**
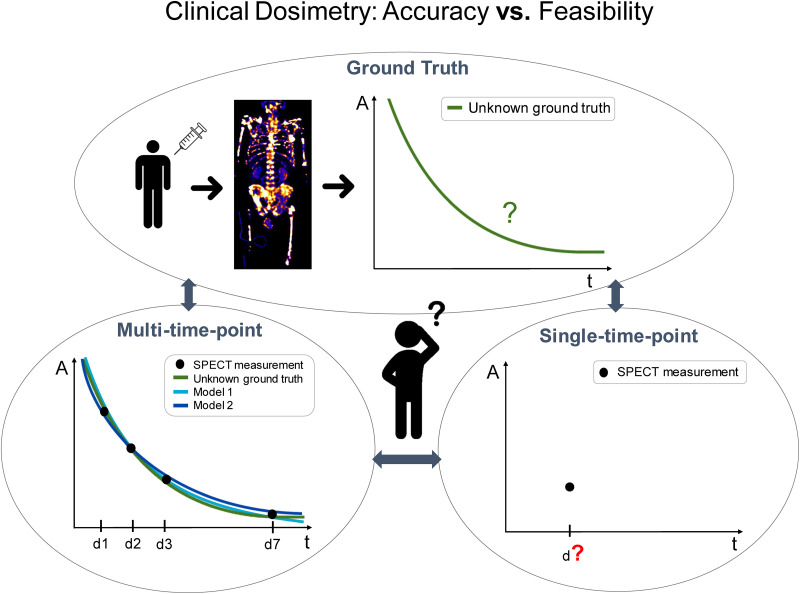

Targeting of the prostate-specific membrane antigen (PSMA) with [^177^Lu]Lu-PSMA therapy evolved as a promising strategy for the treatment of metastasized castration-resistant prostate cancer, which is one of the main worldwide causes of death in men (1). The VISION trial led to the approval of [^177^Lu]Lu-vipivotide tetraxetan (Pluvicto; Novartis) by the U.S. Food and Drug Administration in March 2022 and by the European Medicines Agency in December 2022 (2). Patient-specific internal dosimetry supports safe and efficient patient management during radionuclide therapy by monitoring the absorbed radiation dose to both healthy and malignant tissues (3*,*^4^). However, internal dosimetry requires quantitative images, preferably SPECT or PET, at multiple time points (MTPs) at least over the initial days after injection (5–7). The associated high number of examinations poses a high workload and additional costs to the clinics in cases of unclear reimbursement. These factors have so far limited the routine implementation of clinical dosimetry, although it is more and more legally requested (8*,*^9^). The rising number of therapies as expected after the U.S. Food and Drug Administration and European Medicines Agency approval of [^177^Lu]Lu-vipivotide tetraxetan will probably further complicate the integration of routine dosimetry into the clinical workflow. On the other hand, only routine dosimetry with reasonable accuracy can provide the empiric knowledge that is necessary to tailor PSMA therapy more to the individual patient characteristics, as for example, with respect to treatment activity per cycle or number of cycles.

To handle the workload associated with increasing routine use of dosimetry, dosimetry based on fewer image acquisitions and even single-time-point (STP) dosimetry methods are being intensively investigated for ^177^Lu-based treatments (10–25). Depending on the selected imaging time point, these protocols lay within an acceptable agreement of around 10% compared with a selected ground truth protocol that is based on serial quantitative imaging. To derive the time-integrated activity (TIA) for each tissue of interest, most of the proposed STP methods measure the uptake per tissue based on a single quantitative image and combine this single uptake measurement with some prior knowledge about the tissue-specific effective half-life. The latter could be a population-based effective half-life or the patient-specific effective half-life of a former therapy cycle. Besides the exact STP method used for the calculation of the TIA itself, so far published methods differ regarding the considered reference model (Table 1). The reference model for MTP dosimetry is defined by the number and timing of the image acquisition itself and the mathematic fit model that is applied to the data points. These reference protocols are highly variable between institutions, as internal dosimetry still lacks standardization, and as site-specific protocols must be tailored to the local situation. Because the reference model is usually already limited by clinical feasibility, it cannot be considered a real ground truth but already represents an approximation with some protocol-dependent error. Thus, findings on STP dosimetry such as optimum time point and percentage deviation compared with MTP dosimetry cannot necessarily be transferred from one considered MTP reference protocol to another. The aim of this study was to investigate how the choice of reference model affects selection of the optimum method for STP dosimetry in the scope of [^177^Lu]Lu-PSMA-I&T therapy.

**TABLE 1.** Overview of STP Dosimetry for [^177^Lu]Lu-PSMA Therapy

Study	Compound	Reference time points	Reference fit	Optimum STP (h)			Criterion for optimal STP
				Kidneys	Lesions	Salivary glands	
Brosch-Lenz et al. (10)	[^177^Lu]Lu-PSMA-617	24, 48, 72 h	Monoexponential	48	72	NA	Percentage difference
Jackson et al. (11)	[^177^Lu]Lu-PSMA-617	4, 24, 96 h	Triexponential	<48	≥72	<48	Mean absolute deviation
Rinscheid et al. (18)	[^177^Lu]Lu-PSMA-I&T	30–120 min, 24 h, 168 h	PBPK/monoexponential	52	72 h	NA	Root-mean-squared error
Peters et al. (17)	[^177^Lu]Lu-PSMA-617	1, 24, 48, 72, 168 h	Compartment-specific combination of linear interpolation and monoexponential	24/48	168	24/48	Various (e.g., Lin concordance correlation coefficient)
Kurth et al. (13)	[^177^Lu]Lu-PSMA-617	2, 24, 48, 72 h	Rapid uptake + biexponential	48	—	48	Bland–Altman analysis, rmANOVA

NA = not applicable; PBPK = physiologically based pharmacokinetics; rmANOVA = repeated-measures ANOVA.

## MATERIALS AND METHODS

### Patients

This study included 7 patients diagnosed with metastasized castration-resistant prostate cancer in their first cycle of [^177^Lu]Lu-PSMA-I&T therapy (Table 2). All patients gave written consent to undergo radiopharmaceutical therapy. The institutional review board approved this retrospective study, and the requirement to obtain informed consent was waived (reference 22-0552).

**TABLE 2.** Patient Information

Patient no.	Age (y)	Weight (kg)	Height (cm)	PSA (ng/mL)	Activity (GBq)	Number of lesions in field of view	Location of segmented lesions
1	68	83	173	84	7.40	7	Bone
2	83	58	166	197	7.45	8	Bone
3	68	95	180	286	7.39	4	Lymph node
4	83	84	187	551	7.43	9	Bone
5	76	75	170	1,500	8.93	15	Bone
6	55	68	170	4.6	7.94	4	Bone
7	68	85	183	52	7.84	13	Bone

### Imaging

SPECT/CT measurements were scheduled 24, 48, 72, and 168 h after injection on a dual-head Symbia Intevo T16 SPECT/CT (Siemens Healthineers) using a standard ^177^Lu protocol (26) (Supplemental Fig. 1; supplemental materials are available at http://jnm.snmjournals.org).

### Dosimetry

Sixty lesions, 14 kidneys, and 10 submandibular glands were delineated daywise in the SPECT/CT data. Kidney volumes of interest (VOIs) were drawn manually on the CT data. Lesions and submandibular glands were delineated using a 30% isocontour of the average within a spheric VOI of 12-mm diameter centered at the tissue maximum (26*,*^27^). To allow for a more robust quantification, only lesions with volumes larger than 5 cm^3^ were included. Only 10 submandibular glands could be investigated since segmentation was not possible for 2 patients because the field of view was too small. The parotid glands could not be evaluated, as they were not completely covered in the SPECT acquisitions. Mean activity concentrations and volumes were extracted. Absorbed doses were estimated using an in-house routine and mass-scaled organ and tumor S values as extracted from OLINDA/EXM version 2.0 (lesions: 2.33e−05 Gy/[MBq × s], 1 g; kidneys: 7.74e−08 Gy/[MBq × s], 310 g; salivary glands: 2.76e−07 Gy/[MBq × s], 85 g) (28).

### Reference MTP Dosimetry

The TIA was first estimated for each compartment by fitting and integrating all available imaging time points using a monoexponential model and a biexponential model. For the latter, all possible realizations with up to 3 free parameters as proposed by Hardiansyah et al. were considered (29). All fit functions were evaluated by common goodness-of-fit criteria, that is, the coefficients of variation (CV) and the sum of squared errors (30*,*^31^). Finally, the following 2 functions were used to fit the reference MTP model:

$$f_{\text{mono}}\left(t\right)=A_{0}e^{-\left({\text{λ}}_{\text{bio}}+{\text{λ}}_{\text{phys}}\right)t},$$

$$f_{\text{bi}1,\text{sγ}}\left(t\right)=A_{0}\text{γ}e^{-\left({\text{λ}}_{\text{bio}}+{\text{λ}}_{\text{phys}}\right)t}+A_{0}\left(1-\text{γ}\right)e^{-\left({\text{λ}}_{\text{phys}}\right)t}\;\text{with}\;\text{shared}\;\text{γ}.$$

*A*_0_ refers to *A*(*t* = 0), and ${\text{λ}}_{\text{bio}}$ and ${\text{λ}}_{\text{phys}}$ are the biologic and physical clearance rates, respectively. The population-based γ-parameter modulates the transition from the phase with biologic clearance to the phase without biologic clearance and was determined by the jackknife method. For the latter, the respective function is fitted to the data of all patients except the one currently being investigated. The CV refer to the 2 free parameters *A*_0_ (CV_A_) and λ_bio_ (CV_λ_).

### STP Dosimetry

Three different STP approaches were evaluated with a single SPECT/CT acquisition at 24, 48, 72, or 168 h after injection: a monoexponential (STP_mono_) or a biexponential (STP_bi_) function with population-based biologic clearance rates; the method by Hänscheid et al., originally established for ^177^Lu-DOTATE/DOTATOC therapy (STP_H_) (12); and the method proposed by Jackson et al. (STP_J_) (11).

The TIA for STP_mono_ is given by

$${\text{TIA}}_{\text{mono}}=\frac{1}{\left({\text{λ}}_{\text{bio}}+{\text{λ}}_{\text{phys}}\right)}\;\frac{A\left(t_{\text{SPECT}}\right)}{\exp\left(-({\text{λ}}_{\text{bio}}+{\text{λ}}_{\text{phys}}\right)\cdot t_{\text{SPECT}})}.$$

$t_{\text{SPECT}}$ and $A\left(t_{\text{SPECT}}\right)$ refer to imaging time point and the VOI activity measured for that time point, respectively. Two population-based parameters, ${\text{λ}}_{\text{bio}}$ and γ, were used for STP_bi_, with the TIA being given by

$${\text{TIA}}_{\text{bi}}=\frac{A\left(t_{\text{SPECT}}\right)}{\text{γ}\;\text{exp}\left(-({\text{λ}}_{\text{bio}}+{\text{λ}}_{\text{phys}}\right)\cdot t_{\text{SPECT}})+\left(1-\text{γ}\right)\cdot \text{exp}\left(-{\text{λ}}_{\text{phys}}\;t_{\text{SPECT}}\right)}\cdot \left(\frac{\text{γ}}{{\text{λ}}_{\text{bio}}+{\text{λ}}_{\text{phys}}}+\frac{1-\text{γ}}{{\text{λ}}_{\text{phys}}}\right).$$

For STP_H_, the TIA is estimated by

$${\text{TIA}}_{\text{H}}=A\left(t_{\text{SPECT}}\right)\cdot \frac{2}{\ln\left(2\right)}\cdot t_{\text{SPECT}\;}.$$

Hänscheid et al. stated that if imaging is performed between $0.75\;t_{1/2,\text{eff}}$ and $2.5\;t_{1/2,\text{eff}}$ ($t_{1/2,\text{eff}}$ is effective half-life), the error in the TIA is less than 10% (12). In this study, we investigated all available time points, assuming that time points that do not fulfil this criterion will automatically show a larger deviation from the reference. More information about the VOIs and time points $t_{\text{SPECT}}$ for which the criterion $0.75\;t_{1/2,\text{eff}}$ < $t_{\text{SPECT}}$ < $2.5\;t_{1/2,\text{eff}}$ was applied is given in Supplemental Table 2.

For STP_J_, it is assumed that

$${\text{TIA}}_{\text{J}}=A\left(t_{\text{SPECT}}\right)\cdot f_{\text{tissue}}\left(t_{\text{SPECT}}\right),$$

with *f*_tissue_ being a scaling factor depending on time point and compartment. The factors were recalculated for our patient cohort according to the method described by Jackson et al. because of the different PSMA compound used in this study (Supplemental Table 2) (11).

### Data Evaluation

The Wilcoxon signed-rank test was used to compare the dosimetry estimates and the goodness-of-fit parameters for the reference models monoexponential MTP (MTP_mono_) and biexponential MTP (MTP_bi_). Mean absolute deviations of STP methods from MTP_mono_ and MTP_bi_ were determined per compartment, whereupon STP_mono_ was compared only with MTP_mono_ and STP_bi_ only with MTP_bi_. The best imaging time point or STP method was defined as the one with the lowest mean absolute deviation. The percentage of VOIs with a deviation of less than |10|% was used as a secondary criterion.

## RESULTS

Table 3 shows the goodness-of-fit parameters for both the mono- and biexponential reference model, as well as the statistical evaluation. For the salivary glands, MTP_bi_ seemed to be superior to MTP_mono_, as both the CV and the sum of squared errors were significantly lower (*P* < 0.05). Such was not the case for the kidneys. The average goodness-of-fit parameters would favor MTP_bi_ but without statistical significance (*P* > 0.05). For the lesions, CV λ_bio_ significantly favored MTP_mono_ (*P* < 0.05), whereas the sum of squared errors significantly favored MTP_bi_ (*P* < 0.05). However, the monoexponential reference model yielded significantly lower absorbed doses than did the biexponential fit (Table 3). Although for both the kidneys and the lesions, no reference model was deemed to be superior, maximum absorbed dose deviations of −19% and −44% were found for kidneys and lesions, respectively (Table 3). As expected, CV λ_bio_ was found to be larger for lesions than for kidneys and salivary glands, indicating a larger pharmacokinetic variability for lesions. Average (±SD) effective half-lives were found to be 56.5 ± 18.8, 32.9 ± 5.9, and 22.5 ± 2.8 h for lesions, kidneys, and salivary glands, respectively. More information on the distribution of effective half-lives and some exemplary time–activity curves are shown in Supplemental Figures 3–5.

**TABLE 3.** Fit and Dosimetry Results for Reference Models

Parameter	Kidneys (*n* = 14)	Salivary glands (*n* = 10)	Lesions (*n* = 60)
Volume ± SD (cm^3^)	216 ± 58	10 ± 2	25 ± 25
MTP_mono_ goodness of fit			
CV_A_/CV_λ_ (%)	3.3 ± 2.3/6.3 ± 5.3	11.1 ± 7.6/14.9 ± 9.1	8.3 ± 4.5/25.8 ± 18.6
SSE	6.2 ± 5.6	0.05 ± 0.02	8.3 ± 17.0
Dose ± SD* (Gy/GBq)	0.32 ± 0.07	0.21 ± 0.08	0.95 ± 0.50
Half-life ± SD (h) (CV)	32.9 ± 5.9 (17.8%)	22.5 ± 2.8 (12.3%)	56.5 ± 18.8 (33.2%)
MTP_bi_ goodness of fit			
CV_A_/CV_λ_ (%)	3.1 ± 2.2/6.0 ± 5.1	8.2 ± 8.1/9.9 ± 7.0	8.9 ± 6.0/28.3 ± 22.2
SSE	3.8 ± 3.2	0.01 ± 0.01	6.4 ± 12.0
Dose ± SD^†^ (Gy/GBq)	0.36 ± 0.09	0.28 ± 0.1	1.10 ± 0.70
Comparison of calculated absorbed dose: MTP_mono_ vs. MTP_bi_			
AD ± SD^†^ (%)	−10.6 ± 3.8	−24.2 ± 3.1	−13.9 ± 9.7
Maximum deviation^†^ (%)	−19	−28	−44
* P* value^‡^	<0.001	0.002	<0.001
Comparison of goodness-of-fit parameters: MTP_mono_ vs. MTP_bi_			
* P* values^‡^ CV_A_/CV_λ_	0.583/0.761	0.027/0.020	0.233/0.026
* P* values^‡^ SSE	0.135	0.002	<0.001

* Values without recovery correction.

† Calculated via $\frac{\left({\text{MTP}}_{\text{mono}}-{\text{MTP}}_{\text{bi}}\right)}{{\text{MTP}}_{\text{bi}}}\cdot 100\text{\%}$.

‡ Wilcoxon signed-rank test.

CV_A_/CV_λ_ = CV A_0_/CV λ_bio_; AD ± SD = average deviation ± SD of absorbed dose; SSE = sum of squared errors.

The deviations of all STP methods and imaging time points from the reference models are listed in Supplemental Tables 3 and 4.

### Kidneys

**FIGURE 1.**
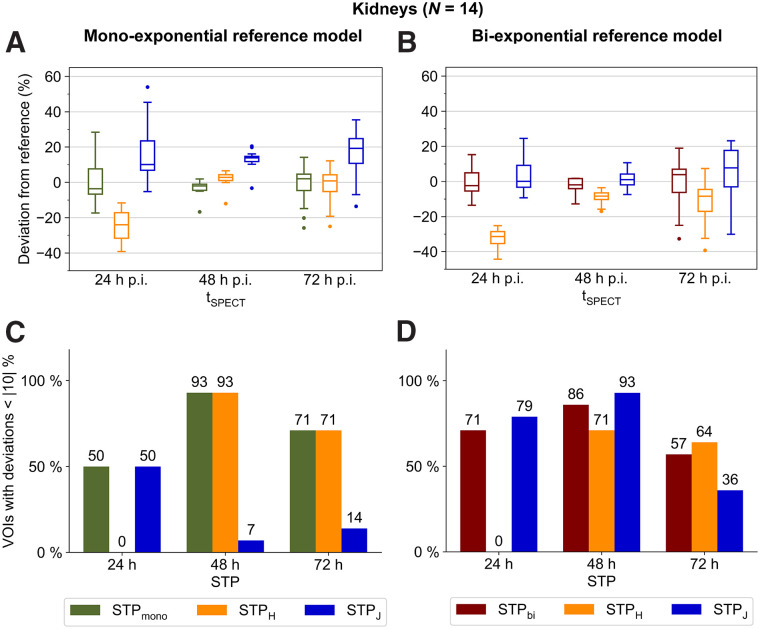
(A and B) Deviation of kidney STP dosimetry from MTP dosimetry using monoexponential (A) or biexponential (B) MTP reference models and for different imaging time points. (C and D) Percentage of VOIs with deviation less than |10|% using monoexponential (C) or biexponential (D) MTP reference models and for different imaging time points. Numbers above each bar indicate exact percentages. For better visualization, data for 168 h after injection are shown in Supplemental Figure 6 and Supplemental Tables 5 and 6. p.i. = after injection.

For both reference models, 48 h after injection was found to be the optimum imaging time point (Figs. 1A and 1B). STP_mono_ and STP_H_ showed the smallest mean absolute deviations from the monoexponential reference model MTP_mono_, at 3% ± 4% and 4% ± 4%, respectively. Compared with the biexponential reference model MTP_bi_, STP_bi_ and STP_J_ indicated the smallest mean absolute deviations of 4% ± 4% and 4% ± 5%, respectively, with a slight preference for STP_bi_ since for 86% (vs. 71%) of VOIs the renal absorbed doses deviated less than 10% from the reference (Fig. 1D).

### Salivary Glands

**FIGURE 2.**
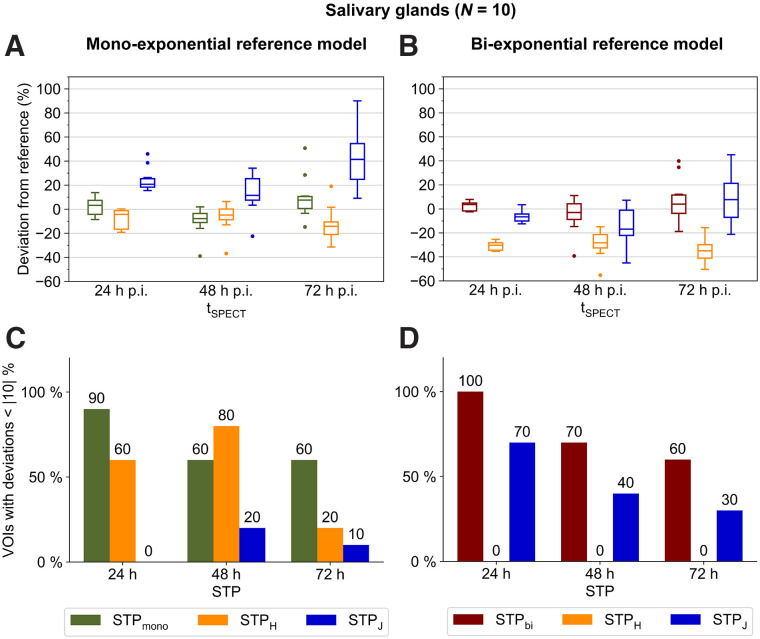
(A and B) Deviations of salivary gland STP dosimetry from MTP dosimetry using monoexponential (A) and biexponential (B) reference models and different imaging time points. (C and D) Percentage of VOIs with deviation less than |10|% using monoexponential (C) and biexponential (D) reference models and different imaging time points. Numbers above each bar indicate exact percentages. For better visualization, data for 168 h after injection are shown in Supplemental Figure 6 and Supplemental Tables 5 and 6. p.i. = after injection.

SPECT/CT imaging at 24 h after injection in combination with STP_bi_ resulted in the lowest mean absolute dose deviation of 4% ± 4% (all VOIs within |10|%), when compared with the best-suited reference model, that is, MTP_bi_ (Figs. 2B and 2D). At 48 h after injection, STP_bi_ showed the best performance as well (mean absolute deviation of 10% ± 14%, with 70% of all VOIs within |10|%) (Figs. 2B and 2D).

### Lesions

**FIGURE 3.**
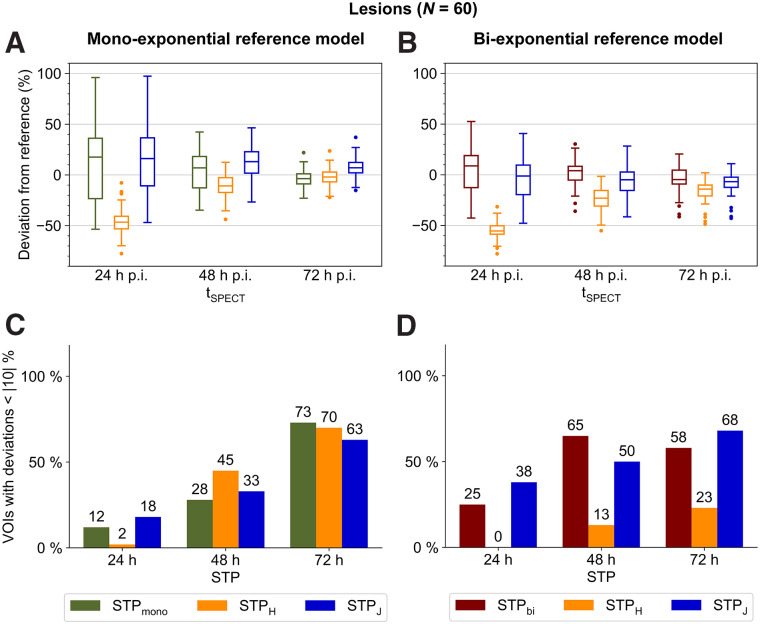
(A and B) Deviation of lesion STP dosimetry from MTP dosimetry using monoexponential (A) or biexponential (B) MTP reference models and for different imaging time points. (C and D) Percentage of VOIs with deviation less than |10|% using monoexponential (C) or biexponential (D) MTP reference models and for different imaging time points. Numbers above each bar indicate exact percentages. For better visualization, data for 168 h after injection are shown in Supplemental Figure 6 and Supplemental Tables 5 and 6. p.i. = after injection.

Larger deviations from the reference models were observed for lesions than for at-risk organs, with 48–72 h being the best-suited time point (Figs. 3A and 3B). Compared with MTP_mono_, STP_mono_ and STP_H_—both with imaging at 72 h after injection—showed a similar performance (average deviation, 8% ± 9% and 7% ± 9%, respectively, with 73% and 70% of lesions deviating less than 10% from MTP_mono_) (Figs. 3A and 3C). Compared with MTP_bi_, similar average deviations for all 3 STP methods could be obtained (STP_bi_, 10% ± 13%; STP_H_, 16% ± 10%; and STP_J_, 10% ± 10%) but with use of different optimum imaging time points (72 h for STP_H_ and STP_J_, 48 h for STP_bi_) (Figs. 3B and 3D).

## DISCUSSION

The aim of this study was to compare different STP approaches for 2 reference models and—apart from the bone marrow—the main compartments of interest during [^177^Lu]Lu-PSMA treatment, that is, lesions, kidneys, and salivary glands. Although MTP_bi_ could be identified as the best-suited reference model for the salivary glands, no superiority of MTP_bi_ or MTP_mono_ could be established for kidney and lesion dosimetry using SPECT acquisitions at 24, 48, 72, and 168 h after injection. However, absorbed dose estimates were found to be significantly different for both reference models. A higher number of image acquisitions may help to reduce the ambiguity of the optimum reference model for lesion and kidney dosimetry. Yet, the number of image acquisitions available in a clinical setting is inherently limited. For example, although the late pharmacokinetics has a significant impact on dosimetry estimates (32), image acquisitions are usually terminated, at the latest, at around 7–9 d after injection for logistic reasons and because SPECT imaging at late time points is limited by a decreasing activity concentration. Although STP dosimetry is feasible, Figures 1, 2, 3A, and 3B highlight that the error of each STP method depends on the reference MTP protocol. Thus, an uncertainty in the reference MTP protocol automatically translates into the selection of the optimum STP method. Comparability of STP dosimetry from different studies requires careful consideration of the reference protocols that were used to define the STP methodology. In this sense, the popularity of STP dosimetry also strengthens the request for standardization of clinically feasible but reliable MTP protocols.

The optimum imaging time point for renal STP dosimetry is clearly 48 h for all STP methods and both reference models. As no superiority for MTP_bi_ or MTP_mono_ could be proven and, thus, the underlying pharmacokinetics is not well known, STP_mono_ and STP_bi_ should be avoided. Thus for kidney STP dosimetry, STP_H_ is recommended over STP_J_ because the number of VOIs with a deviation of less than 10% is high for both MTP_mono_ and MTP_bi_. Similarly, STP_J_ with imaging at 72 h can be recommended for lesion STP dosimetry. For the salivary glands, MTB_bi_ could be identified as the optimum reference model, with imaging at 24 h after injection and STP_bi_ being the best choice for STP dosimetry.

The definition of a specific STP methodology is further complicated by the fact that the optimal STP dosimetry for different compartments is usually associated with different optimum imaging time points. From a clinical perspective, STP approaches should be available with similar performance for lesions and organs at risk. However, considering the results in both Figure 1 and Figure 2, compared with Figure 3, a trade-off between the accuracy of dosimetry for lesions and at-risk organs is necessary. The optimum time point is also likely to depend on the question of whether the primary objective of dosimetry is safety or efficacy. It may be possible to merge STP dosimetry for lesions, kidneys, and salivary glands based on imaging at 48 h after therapy, although this will result in a lower level of accuracy for lesions (optimum, 72 h after injection) and salivary glands (optimum, 24 h after injection). For the salivary glands with MTP_bi_ as the optimum reference protocol, STP_bi_ is suggested for imaging at 48 h after injection. For lesion dosimetry, STP_J_ is recommended for imaging at 48 h after injection, as it shows a more balanced performance for MTP_bi_ and MTP_mono_ than does STP_H_.

The overall number of patients in this study was low. Especially, the used biexponential reference model applies a population-based shared parameter γ that should be reevaluated for a larger patient cohort. Further, this population-based parameter may vary over the course of subsequent therapy cycles, whereas only the first therapy cycle was investigated in this study. However, Hardiansyah et al. found a value of 0.963 ± 0.004 for the renal biokinetics of 13 [^177^Lu]Lu-PSMA-I&T patients (29), compared with a very similar value of 0.956 ± 0.007 (CV, 0.7%) for 14 kidneys and 7 patients in this study (0.8% deviation). In addition, the method proposed by Hänscheid et al. (12) was originally developed for monoexponential pharmacokinetics but was compared with a biexponential reference model in this study as well. However, the intention was to show the effect of an unknown ground truth model on STP methodology. For example, for the salivary glands, with clearly biexponential pharmacokinetics, STP_H_ showed poor performance. Despite the smaller patient cohort, the optimum time points proposed in this study agreed well with previously published data (10*,*^11^*,*^13^*,*^17^*,*^18^). As data with a late SPECT scan at around 7 d after injection were available for only a selected number of patients undergoing their first treatment cycle in this study, no prior information on the compartment-specific effective half-life was available, limiting this study to population-based approaches only. Brosch-Lenz et al. investigated a prior-information approach based on the patient-specific effective half-life of the previous treatment cycle in comparison to the method proposed by Hänscheid et al. for kidney and lesion STP dosimetry and for [^177^Lu]Lu-PSMA-I&T therapy (10*,*^12^). The reference model was a monoexponential fit with SPECT/CT imaging at 24, 48, and 72 h after treatment. The method by Hänscheid et al. was slightly superior for the kidneys, whereas for lesion STP dosimetry the prior-information approach showed better performance, possibly because the lesion pharmacokinetics usually shows a larger interpatient and intrapatient variability.

Population-based approaches assume an average patient with average pharmacokinetics. It should be further investigated whether patient-specific factors, such as changes in tumor volume, kidney function, or any other patient-specific factors that deviate from the average patient, may guide the decision between MTP and STP dosimetry. Scheduling MTP dosimetry intermittently (e.g., alternating between MTP and STP dosimetry) may be a trade-off between clinical efficiency and accuracy. Further, in view of the observed outliers for different STP approaches, quality control is an important aspect of STP dosimetry. The use of mixed-linear-effects models, which consider each parameter as a combination of a mixed population-based effect and a random patient-specific effect, has already been shown to be promising for reducing outliers within STP dosimetry (19*,*^24^).

Current research also focuses on speeding the SPECT acquisition, a development that is lately being driven by the integration of artificial intelligence into molecular imaging (33–35). This should be considered as an opportunity to maintain MTP-based protocols, at least in centers that hospitalize patients or where multiple examinations are feasible on an outpatient basis.

## CONCLUSION

STP dosimetry is becoming more popular because it allows for efficient integration of internal dosimetry into clinical routine. Usually, however, the considered reference MTP protocol already approximates the real but unknown clinical ground truth, and its error is in addition to the error of STP dosimetry itself. The intercomparison of different STP methods shows a different optimum time point and error depending on the compartment and the reference MTP protocol. According to our patient cohort, 48 h after injection may be regarded as a compromise for STP dosimetry for lesions and at-risk organs.

## DISCLOSURE

Astrid Delker is partially funded by the Federal Ministry of Education and Research (funding number 02NUK065C). No other potential conflict of interest relevant to this article was reported.

## Notes

KEY POINTS **QUESTION:** Does the reference model impact findings on STP dosimetry for [^177^Lu]Lu-PSMA-I&T therapy? **PERTINENT FINDINGS:** The error of each STP dosimetry method depends on compartment, time point, and reference MTP protocol. In our patient cohort, 48 h may be regarded as a compromise for dosimetry for lesions and healthy tissues. **IMPLICATIONS FOR PATIENT CARE:** STP dosimetry for [^177^Lu]Lu-PSMA-I&T can be performed, but at the cost of some level of accuracy. Further efforts to standardize internal dosimetry are important to harmonize MTP and STP dosimetry among centers.
